# Protective role of adjuvant and potassium permanganate on oxidative stress response of Nile tilapia (*Oreochromis niloticus)* challenged with *Saprolegnia ferax*

**DOI:** 10.1186/2193-1801-2-94

**Published:** 2013-03-09

**Authors:** Eman Zahran, Engy Risha

**Affiliations:** Department of Internal Medicine, Infectious and Fish Diseases, Faculty of Veterinary Medicine, Mansoura University, El-Gomheria Street, Mansoura, Egypt; Department of Clinical Pathology, Faculty of Veterinary Medicine, Mansoura University, El-Gomheria Street, Mansoura, Egypt

**Keywords:** Adjuvant, Oxidative response, Potassium permanganate, *Saprolegnia*, Fish

## Abstract

Saprolegniosis are one of the most important oomycetes affecting freshwater fish worldwide. It leads to huge losses in fish farms due to their massive destruction to the fish epidermis leading to sever heamodilution and death, yet little literature studied the oxidative stress response regarding the saprolegniosis. Nile tilapia fish were subcutaneously abraded and divided into four groups: control group, abraded but not challenged, infected group, challenged with *Saprolegnia. ferax* (*S. ferax*) zoospores 2 × 10^4^ for one week, potassium permanganate (KMnO_4_) group, challenged with *S. ferax* zoospores 2 × 10^4^ for one week then treated with KMnO_4_ and lasted for 2 weeks after, Freund’s complete adjuvant (FCA) group challenged with *S. ferax* zoospores 2 × 10^4^ for one week then treated with FCA and lasted for 2 weeks after and control positive group, challenged with *S. ferax* zoospores 2 × 10^4^ for 3 weeks. Higher mortalities were recovered from the challenged group, which declined upon treatment compared to the continued increased rate in the control positive group. Oxidative stress indicators Nitric Oxide (NO), glutathione (GSH) and superoxide dismutase (SOD) were measured; biochemical parameters: total protein, albumin, globulin, aspartate aminotranseferase (AST), and alanine aminoranferase (ALT) were also measured. Sodium (Na^+^) and potassium (K^+^) levels were measured as indicators of Plasma osmolality. Almost of the measured parameters showed varying reduction significant levels in treatment groups compared with the infected and the control ones. Thus, this study revealed that KMnO_4_ have a protective role against oxidative stress response, furthermore our data provide evidence for the role of FCA in modulating the oxidative stress response and enhancing fish immune response against infections.

**Electronic supplementary material:**

The online version of this article (doi:10.1186/2193-1801-2-94) contains supplementary material, which is available to authorized users.

## Background

The fish pathogenic oomycetes, especially *Saprolegnia* spp; a member of the family *Saprolegnia* ceae, causing major outbreaks in fish and fish hatcheries as well, these infections are widespread and occurred at any stage of fish life cycle (Hussein et al. 2001). Saprolegniosis characterized by a relatively superficial, cottony/woolly (floccous), white growth on the skin, or gills, or on fish eggs when in water. Initial lesions are often focal, small, and inconspicuous, but these can rapidly enlarge because of the rapid development of the mycelium over a short period of time. Lesions may extend into dermis and the subjacent superficial musculature with time with sloughing of the epidermis (Van West 2006).

Many stressors such as adverse water temperature, poor water quality, handling, or crowding are frequently associated with outbreaks of saprolegniosis (Bailey 1984); (Copland & Willoughby 1982); (Whisler 1997). Epithelial damage on the skin, gills, and gut, due to trauma or other pathogens, can provide a route of entry for oomycetes (Roberts 2001). Signs of disease and mortalities were culmination of two related factors: (I) rapid decreases in water temperature from ~20 to 10°C in 24 h induced subsequent immunosuppression; and (2) maintenance of low temperatures favoured high levels of *Saprolegnia* sp. , zoospores (≥5 spores ml) (Bly et al. 1992).

Reactive oxygen species (ROS), such as superoxide anion radicals, hydrogen peroxide, and hydroxyl radicals, are continuously formed in oxygen-consuming organisms. Presence of toxic chemicals or pollutant or infection in fish environment could help in production of these ROS and so create an imbalance and it can result either in subsequent down regulation of the antioxidative response or oxidative damage outright in organisms (Valavanidis et al. 2006). Oomycetes infection in fish produce damage to the epidermis with mycelial penetration, thus fish like other vertebrates, defend their self-using the antioxidant defense system (Alvarez et al. 2005).

Treatment of water mold infection is very difficult and legally available drugs are also limited. Potassium permanganate, copper sulfate and formalin have been used for treatment of water mold infection. Furthermore, salt dips are also used to treat water molds and help to counteract the osmotic stress in the infected fish with damage skin (Treves-Brown, 2000; and Straus *et al.*, 2009). Malachite green (MG) has consistently been the most effective oomyceticidal among numerous compounds tested widely used to treat water mold infections on fish and their eggs, however; many countries have banned the use of MG in food-fish production because of its risk to human health (Treves-Brown, 2000). Thus, many drugs have had been screened for their anti-water mold activity to try to replace MG. Potassium permanganate has been tested as a fungicide on eggs of several fish species. Effective dosages have been found to vary with the fish species tested.

Immunostimulant are agents used to enhance the nonspecific immune response (Sakai 1999). They include synthetic chemicals, drugs, and various natural products. Adjuvants are usually mixed with specific antigen preparations in order to elevate a specific immune response. However, they can also be used alone to elicit only a nonspecific response. Freund’s adjuvant was one of the first immunostimulant to be used in animals and has been shown to elicit a nonspecific immune response (Olivier et al. 1985; Olivier et al. 1986b; Chen et al. 1998).

Tilapia (*Oreochromis* sp.) is one of the most important cultured freshwater fish worldwide, due to faster growth rate in warm waters, resistance to adverse conditions, relatively low production cost, meat quality and flavour, high protein content, and consumer preference for its phenotype (especially, its attractive colour). In Egypt, Nile tilapia (*Oreochromois niloticus*) is one is one of the most significant species for fish farming and for biological studies as well (Zaitseva et al. 2006).

In Egypt, fish farmers suffered from outbreaks of *Saprolegnia* sis in their farms causing severe damage; affecting eggs hatchability and evoke higher mortalities. Most literature concerned with the pathogenicity of *S. parasitica*; we would like here to investigate the pathogenicity of a pathogenic isolate of *S. ferax*. To our knowledge there is no studies have been conducted to investigate the effect of different treatment methods against the oxidative stress response induced by *Saprolegnia* infection in fish. Thus, to better understand their activities underlying *Saprolegnia* infection, we assessed the effect of two different treatments; potassium permanganate (KMnO_4_) as chemical treatment and Freund’s complete adjuvant (FCA) as chemical synthetic immunostimulant on the induced mortalities and the oxidative stress response as well as biochemical parameters in *Saprolegnia* challenged Nile tilapia, the most important cultured fish in Egypt.

## Results

### Pathogenicity experiment

The result showed that the mortality reached levels of 25, 27.5 and 30% of mortality in and KMnO_4_, FCA, and control positive group, respectively. The mortality declined to 15 and 10% by the second week in KMnO_4_ and FCA groups, respectively. No mortalities were recorded in the second week after treatments for KMnO_4_ and FCA groups. However, the number of dead fish was continued to increase in control positive group peaked up to 92.5% after 3 weeks. No mortalities occurred in the control negative group (Table 1).

**Table 1:** **Nile tilapia (** ***Oreochromis niloticus)*** **mortalities per week, total mortalities (TM) % and survival % out of 40 individuals /group for 3 weeks (1st W, 2nd W and 3rdW) to (2 × 10** ^**4**^ **spore/L) zoospores of** ***S. ferax***

Groups	Mortality%			TM%/group	Survival %
	1st W	2nd W	3rd W		
**KMnO**_**4**_	25	15	0	40	60
**FCA**	27.5	10	0	37.5	62.5
**+ve control**	30	42.5	20	92.5	7.5
**-ve control**	0	0	0	0	100

**Figure 1:**
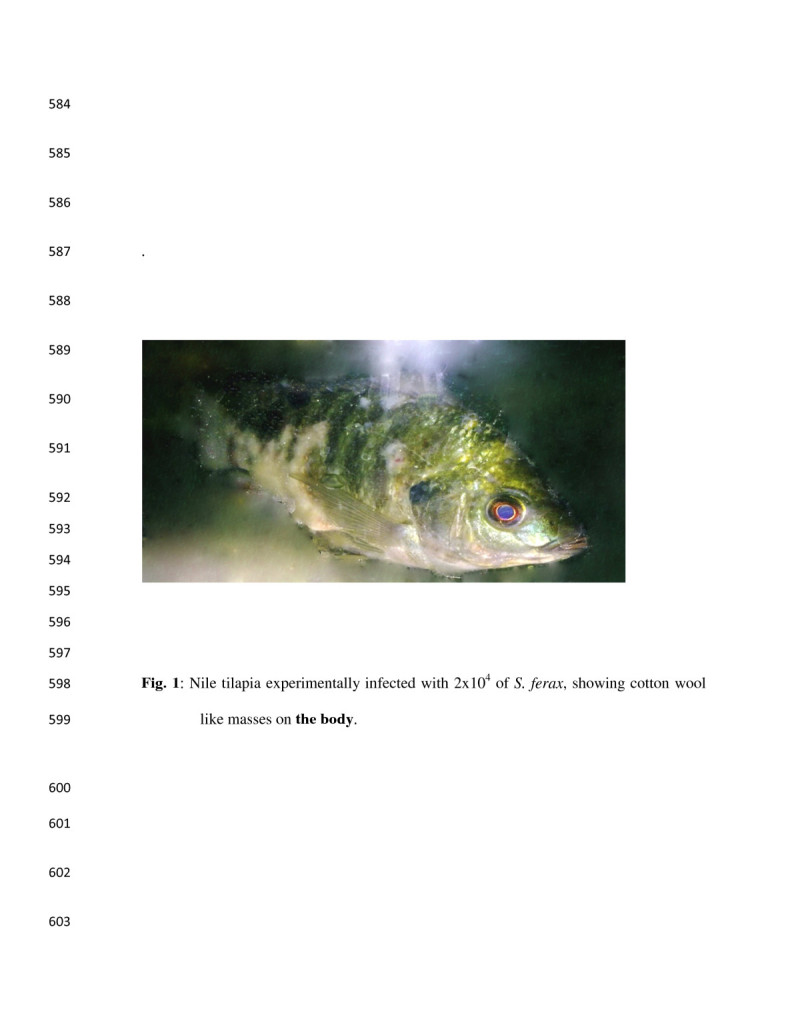
**Nile tilapia experimentally infected with 2x10** ^**4**^ **of** ***S. ferax*** **, showing cotton wool like masses on the body.**

The affected fish had typical signs of water mold infection, with cotton-like growths on the body and fins associated with listlessness, erratic swimming, and rising near water surfaces or resting with their abdomen on the aquarium. All dead fish showed mycelia growth on the body (Figure 1).

### Effect on oxidative enzymes

#### Potassium permanganate (KMnO_4_) treatment group

1. Antioxidant response:
  NO level was significantly increased at week1 in the infected group compared to the control negative group. In KMnO_4_ it significantly increased at week 2 and 3 compared to the control group. However, there were no significant differences in NO level between KMnO_4_ group and infected group (Figure 2a).

**Figure 2:**
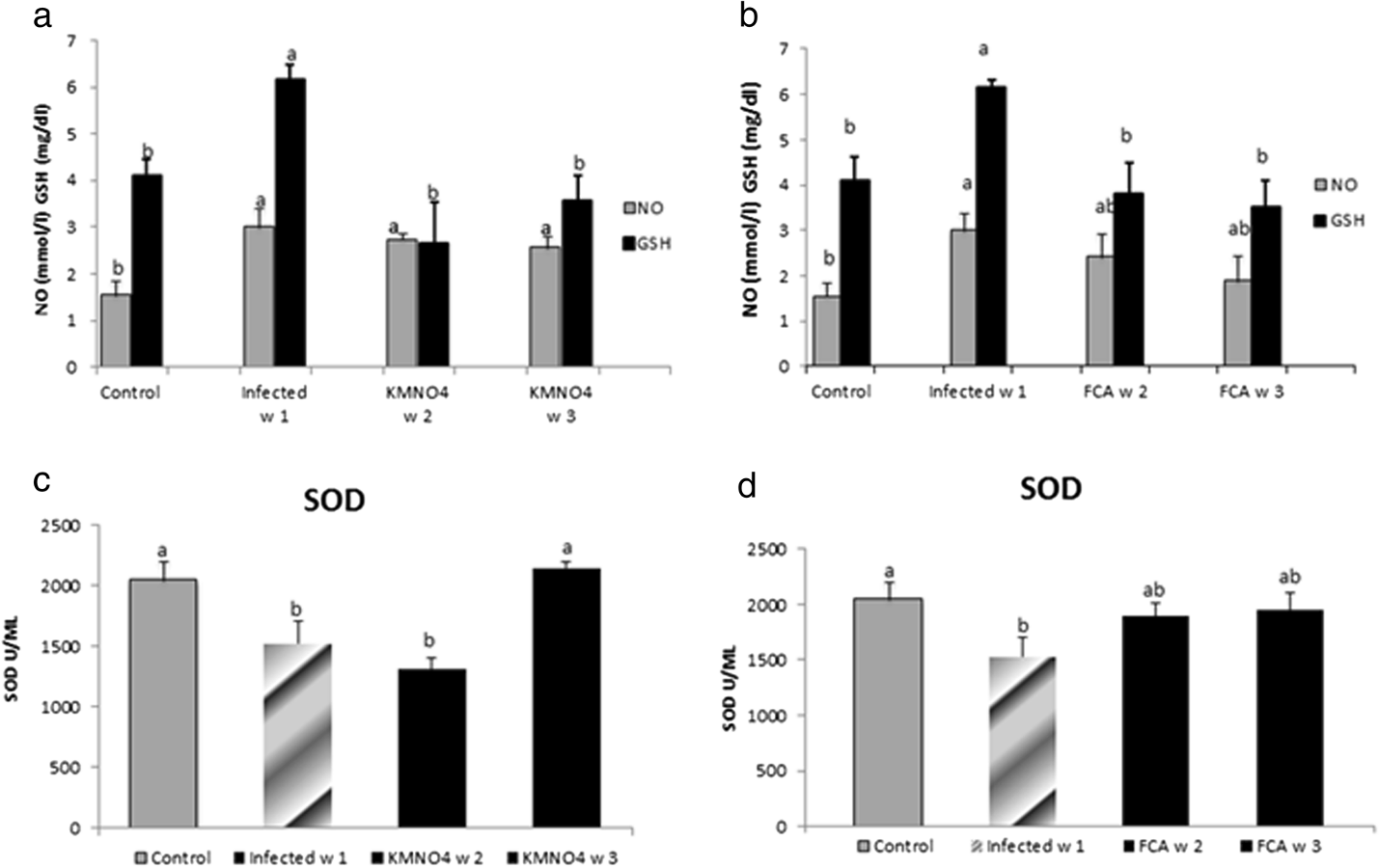
**NO, GSH and SOD response in serum of Nile tilapia (*O. niloticus)*to KMnO**_**4**_**(a, c) and FCA (b, d) treatment group, respectively; compared fish with the infected group challenged with*S. ferax*zoospore 2x10**^**4**^**and control group.** N=8 fish/sampling time. Values are reported as mean ± SE. Values with a different letter superscript are significantly different between and within groups (p < 0.05).
  GSH level was significantly increased at week1 in the infected group in comparison to their control. While at week 2 and 3, the level had a significant a threefold and two fold decreases compared to the infected one; respectively. GSH level was neither significantly different at week 2 and week 3 in KMnO_4_ group nor from the control group (Figure 2a).
  SOD encounter a significant decrease in the infected group at week1 compared to the control where it remained at a lower level at week 2 in KMnO_4_ group. However, SOD had a significant rise again by week 3 after KMnO_4_ treatment to match within control level (Figure 2c).
2. Biochemical analysis and plasma osmolality:
  ALT levels showed no significant changes at any time of experiment. However, AST had a significant rise in the infected group at week 1 which declined by week 2 and 3 in KMnO_4_ group to be within the control level (Figure 3a).

**Figure 3:**
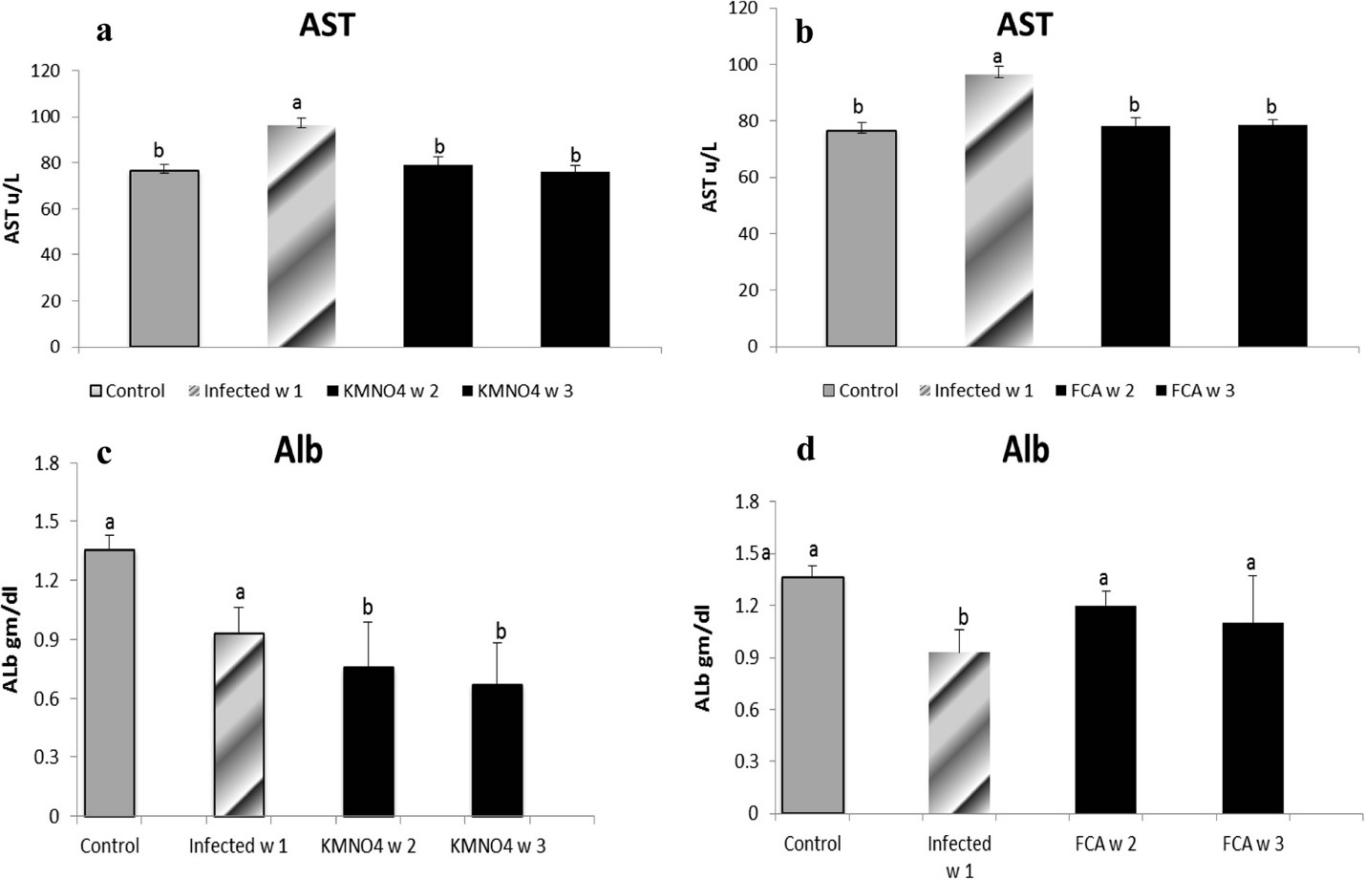
**AST and Albumin levels in serum of Nile tilapia (*O. niloticus)*in KMnO**_**4**_**(a, c) and FCA (b, d) treatment group, respectively, compared fish with the infected group challenged with*S. ferax*zoospore 2x10**^**4**^**and control group.** N=8 fish/sampling time. Values are reported as mean ± SE. Values with a different letter superscript are significantly different between and within groups (p < 0.05).
  Results showed that albumin level had a non-significant nominal decreased at week 1 in the infected group compared to control group, however; in KMnO_4_ group, the level was significantly decreased at week 2 and 3 compared to the infected and the control group (Figure 3c).
  Nile tilapia plasma Na^+^ concentrations had a nominal increase at week 1 in the infected group as well as at week 2 and a significant increase at week 3 in KMnO_4_ group compared to the control group. However, there were no significant differences between both the infected group and KMnO_4_ group. Plasma K^+^ concentration had another trend in which a significant increased level occurred at week 1 in the infected group and week 3 after KMnO_4_ group while it has a non-significant decrease by week 2 in KMnO_4_ group (Figures 4a, 5c).

**Figure 4:**
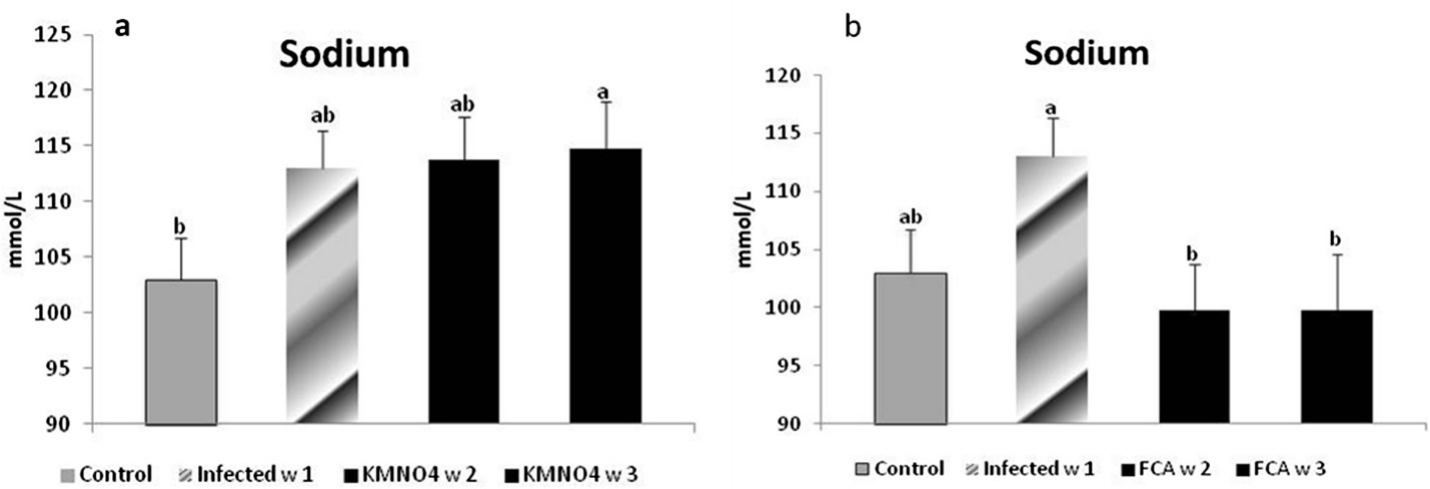
**Sodium level in serum of Nile tilapia (*O. niloticus)*in KMnO**_**4**_**(a) and FCA (b) treatment group, compared with the infected group challenged with*S. ferax*zoospore 2x10**^**4**^**and control group.** N=8 fish/sampling time. Values are reported as mean ± SE. Values with a different letter superscript are significantly different between and within groups (p < 0.05).

**Figure 5:**
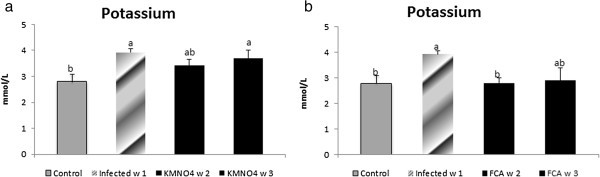
**Potassium level in serum of Nile tilapia (*O. niloticus)*in KMnO**_**4**_**(c) and FCA (d) treatment group, compared fish with the infected group challenged with*s. ferax*zoospore 2x10**^**4**^**, and control group.** N=8 fish/sampling time. Values are reported as mean ± SE. Values with a different letter superscript are significantly different between and within groups (p < 0.05).

#### Adjuvant (FCA) treatment group

1. Antioxidant response:
  NO level was significantly increased in the infected group compared to the control one. However, there were no significant differences between the NO level at week 2 and 3 in the FCA group or with any other group. GSH level was the same manner like the KMnO_4_ group (Figure 2b). SOD encounter a significant decrease in the infected group compared to the control. The level was increased nominally by week 2 and 3 in FCA group (Figure 2d).
2. Biochemical analysis and plasma osmolality:
  ALT and AST levels exhibited same pattern like the KMnO_4_ group (Figure 3b). Albumin level had a different pattern; it has a significant decrease at week 1 in the infected group. However, it encountered a significant increase at week 2 and 3 in FCA group compared to the infected one and there were no significant between both times and the control one (Figure 3d).
  Nile tilapia plasma Na^+^ concentrations had a nominal increased in the infected group at week 1. However, its level had a significant decrease again by week 2 and 3 in FCA group in comparison to both infected and control groups. Plasma K^+^ concentration significantly increased at week 1 in the infected group. At week 2 in FCA group the level decreased again significantly compared to the infected group; but was not significant compared to the control group (Figures 4b, 5d).

## Discussion

Saprolegniosis is one of the most important oomycetes infections that can cause huge economic losses in cultured ecosystems (Van West 2006; Phillips et al. 2008). Our results clearly demonstrated the pathogenicity of *S. ferax* to Nile tilapia associated with higher mortalities. The present results were in agreement with (Stueland et al. 2005) who found that two of seven *Saprolegnia* spp. strains, tested for their pathogenicity to Atlantic salmon *Salmo salar*, caused 89% and 31% cumulative mortality in challenged salmonids. These strains were significantly more pathogenic than the other strains tested. Moreover, (Hussein and Hatai 2002) reported that the cumulative mortalities of the different salmonids fish groups exposed to 2 × 10^5^ spore/L concentrations of *S. salmonis* NJM 9851 were 90% for brown trout, 93.3% for sockeye salmon and 100% for rainbow trout, masu salmon, and Japanese char, however; all salmonid species exposed to 2 × 10^5^ spore/L concentrations of *S. parasitica* NJM 9868 had a cumulative mortalities of 100%. *Saprolegnia* lesions were in form of cottony like masses on different sites on fish body mainly in the dorsal region and on the dorsal and adipose fins (Yanong 2003) which in same event with our results lesion appeared mostly in all sites which are exposed to scarification during descaling process. The death mainly occurred due to the osmotic failure caused by the damaged epidermis (Bruno and Poppe 1996).

Our results showed that Potassium permanganate as a chemical treatment lowering the mortality to 15% in the 2nd week and 0 in the 3rd week; compared to 42.5 and 20% of mortality by 2nd and 3rd week in the control positive group, respectively. The most effective treatment for controlling saprolegniosis is the malachite green; however, since it has several drawbacks (Schreier et al. 1996) it’s not permitted for use in fish-farming in most countries. Thus, the need to search for alternative compounds as effective as malachite green is imperative.

Potassium permanganate is used for ectoparasites, bacteria, fungal disinfections on the skin and gill of fishes, it act as an oxidant through decreasing BOD of water by oxidize the organic matter and the amount of oxygen will be increase by using potassium permanganate (Noga 2010). (Marking et al. 1994) examined two In the same event, (Rasowo et al. 2007) investigated the effect of formaldehyde, sodium chloride, potassium permanganate and hydrogen peroxide treatment for saprolegniosis in catfish (*C. gariepinus)* eggs and found that the best hatchability performance was when eggs treated with 2 ppm potassium permanganate for 30 min (96.7%). (Darwish et al. 2009) evaluate the efficacy of KMnO_4_ at a dose of 2.0 mg/L above the potassium permanganate demand for 2 h duration against Columnaris infection in channel catfish using different route of administration and found that using KMnO_4_ simultaneously with challenge give 99% survival while using KMnO_4_ postchallenge enhanced survival (85%) than positive control (78%); which conclude overall efficacy of KMnO_4_ as a treatment against skin infection. (Marking et al. 1994) reported that potassium permanganate at two concentrations 50 and 100 mg/l decreased fungal infection of rainbow trout eggs but rate of hatching didn’t increase. Our results were supported by (Thomas-Jinu and Goodwin 2004) who found that KMnO_4_ reduced columnaris mortality from 100% to 69%.

Adjuvant treatment as immunostimulant improved the mortality rates from 27.5% to 10% from the first to the second week compared to the control positive which encountered 30% and 42.5% of mortality in week 1and 2. Furthermore, no mortalities were recorded in the 3rd week in FCA group. In addition, FCA treatment showed to some extent a better rate for fish survival compared to KMnO_4_ treatment in the second week after challenge. To our knowledge, there are no studies for using FCA alone as immunostimulant to enhance fish immunity infected with saprolegniosis or investigate its effect regarding the antioxidant response in fish. However, Many co-workers have shown that the adjuvant has a great effect on innate immune response; For example, injection of FCA induces nonspecific protection against several bacterial pathogens and ciliate pathogen of fish (Olivier et al. 1985; Kajita et al. 1992). (Olivier et al. 1985) showed increased resistance of coho salmon (*Oncorhynchus kisutch*) to *Aeromonas salmonicida* after injection of the same preparation of FCA that we used in our studies (i.e., with killed *Mycobacterium butyricum*). Thus, this augmentation of the immunity reflected on the host response to evade the infection. In the same context, (Harikrishnan et al. 2009) found that a prior administration of a triherbal mixure of medicinal plant compounds, azadirachtin (Az), camphor (Ca) and curcumin (Cu), affected mortality rate positively upon changing with virulent starin of *Aphanomyce invadans*. On the contrary, (Kunttu et al. 2009) evaluate the efficacy of two immunostimulants, yeast b-glucan and b-hydroxy-b-methylbutyrate (HMB) to treat the Fingerling rainbow trout experimentally infected with *Flavobacterium columnaris*, and found that given orally both stimulants enhanced the levels of immune function parameters, but did not improve survival in challenge at any concentration of the stimulants used. Intra peritoneal injection of β-glucan increased parameter values several fold, but no beneficial effect of injected glucan on survival was observed. (Zahran et al. 2012) have found that adjuvant parenteral administration in channel catfish could elect a nonspecific defence by expressing antimicrobial polypeptides histone like protein-1 (HLP-1) at a distant site (skin) HLP-1 known with its potent bactericidal, parasiticidal and funigicidal activity (Robinette et al. 1998).

*Saprolegnia* infection resulted from many stress factors that fish are exposed, these factors lowering its resistance and enhance fish susceptibility to infection, *Saprolegnia* act through secreting proteolytic like enzymes that alter integument integrity and facilitate penetration (Peduzzi and Bizzozero 1977).

Antioxidant activity in our study showed different patterns between both treatments to some extent; but as general both treatment had general same effects regarding the antioxidant response. NO is an important regulating signaling molecule, it produced endogenously from L-arginine and molecular oxygen by the enzyme nitric oxide synthase (Bogdan 2001). NO mediate its biological activity by binding with different targets such as, heme groups, cysteine residues, and iron and zinc clusters, thus a certain regulation of NO production is required to mediate its biological effect. When NO level is too high that may indicate presence of toxicity, stressful conditions, infectious agent that all participate in occurrence of the disease (Farrell et al. 1992; Ignarro 2000; Vladutiu 1995). In the present result, NO level significantly increased in the infected group, while with both treatments they significantly decreased to within the normal value. A similar response was observed in rainbow trout inoculated with a virulent strain of *Renibacterium salmoninarum* compared with avirulent strains (Campos-perez et al. 2000), In the same trend (Acosta et al. 2004) found enhanced response in serum concentrations of stable nitric oxide (NO) metabolites in small weight gilthead sea bream (*Sparus aurata*) (30-75 g body weight) lasting from 6 h to six days post-infection with a peak at 24 h; when inoculated of with a sublethal dose of different *Photobacterium damsela* e subsp. *piscicida* (Pdp) strains (DI-21 and 94/99), however; no such response was detected in larger fish (150-600 g).

GSH is an important antioxidant and the amount of GSH present could reflect the antioxidant potential of an organelle (Liu et al. 2008). GSH activities were significantly higher during the challenge in the infected group. This could be attributed to the induction of Reactive oxygen species (ROS), and so more antioxidant enzymes are released to compensate the damage produced by infection (Di Giulio et al. 1989; Storey 1996). (Wilhelm Filho et al. 2002) found that gill tissues are one of the main contributors of ROS generation in fish. This explanation is supported also by other studies by (Thomas and Wofford 1984; Gallagher et al. 1992; Thomas and Juedes 1992) where they demonstrated higher level of GSH following exposing fish to stressor. (Oliveira et al. 2008) showed a higher adaptive competence expressed as antioxidant defenses activation, namely GSH and glutathione peroxidase (GPX) in Liver against different concentration of Phenanthrene. However, SOD level was significantly reduced upon challenge and that could be due to disruption of erythrocyte membranes by ROS caused hemorrhage and loss of antioxidant enzymes in human immunodeficiency virus (HIV-infected humans); and the typical symptom in grass carp hemorrhage virus (GCHV) infected grass carp is erythrocyte hemolysis and bleeding in the muscle may relate to the significantly decreased SOD and catalase (CAT) activities (Zhang et al. 2003) when the grass carp were infected with GCHV. Interestingly, a significant decrease in SOD was also observed in white spot syndrome virus (WSSV) infected *Penaeus monodon* (Chang et al. 2003). Our results in accordance with (Kim et al. 2009) who examined the effect of β-glucan, an immunostimulatory agent, on the superoxide dismutase (SOD) and catalase (CAT) activities of erythrocytes and Mx gene expression in grass carp challenged with grass carp hemorrhage virus (GCHV) and found that the SOD and CAT activities significantly decreased when the fish were challenged with GCHV, but it was higher in the group pre-treated with β-glucan than in infected. Similar results were obtained in Wister rats when injected mangiferin I/P at different dose to overcome cyclophosphamide toxicity; showing that Cyclophosphamide significantly lowered the superoxide dismutase and catalase (CAT) activities in lymphocytes, polymorphonuclear cells (PMN) and macrophages, while the administration of mangiferin significantly protected the activities of SOD and CAT, demonstrating the antioxidant mechanism to exist in its immunoprotective role.

Thus, it’s evident that the KMnO_4_ and adjuvant a promising tools in a protecting against immunological tissue injury may be through the regulation of antioxidant enzyme activities, thus potentiating the cellular antioxidant capacity. Moreover, it emphasize on the role of adjuvant as an immunoprotective role mediated through the inhibition of reactive intermediate-induced oxidative stress in lymphocytes, neutrophils and macrophages. Adjuvant affect macrophage activity and phagocytosis (Olivier et al. 1986a) and thus the respiratory burst activity due to an increase in the antioxidant oxidation level in phagocytes which is an important indicator of innate immune response (Miyazaki 1998).

Biochemical analysis, revealed changes in level of Albumin, AST but no changes have been found in total protein, globulin or ALT. AST level was markedly increased in the infected group compared to KMnO_4_ and FCA groups, while ALT didn’t change. Others studies were in support to our results, they found that increased activity of AST, CK, and LDH are related to venipuncture, which is done through the musculature of the caudal peduncle. While, Sorbitol dehydrogenase and ALT appear to be present in low concentration in skeletal muscle and may be better indicators of hepatocellular damage and this indicate that there were no substantial damage to internal organ as the liver (Tripathi et al. 2003). Also, Biochemical changes in fish included significant hyperglycemia, hyponatremia, and hypochloridemia were evident in koi (*Cyprinus carpio*)experimentally infected with *Flavobacterium columnare* infection (Tripathi et al. 2005).

Albumin level exhibited a moderate decrease in the infected group compred to the control one, while it showed significant reduction by week 2 and 3 in both KMnO_4_ group, but in FCA the albumin level matched within the control one. This may happen as a result of epidermal damage due to *Saprolegnia* also, through skin ulcers present on skin surface, these can represent a portal of loss of plasma protein or that excess of water diffused to the fish body through these sites resulting in slight heamodilution (Tripathi et al. 2005). Our results were in accordance with (Ruane et al. 2002) who observed reduction in plasma protein levels in common carp after confinement and in red sea bream *Pagrus major* following acute handling stress (Biswas et al. 2006). In salmonids, a reduction in serum protein concentration has been observed when the fish were infected or stressed (Melingen et al. 1995; Møyner et al. 1993). These differences are possibly species-specific effects of environmental conditions on serum/plasma protein and globulin concentrations in fish.

Plasma ion concentration in the present study showed variations in their levels; Na^+^ and K^+^ was increased in the infected group and decreased to some extent in the other groups. This is also can be explained in the same context which account for damaged epidermis and loss of the integument integrity and so increase in the permeability with loss of this ions or being diffused from water into fish body. Changes in biochemical parameters in hybrid tambacu fish naturally parasitized by *Dolops carvalhoi* (*Crustacea, Branchiura*), a fish louse were in form of increases in MCHC, plasma glucose levels, serum protein, sodium and chloride levels, number of monocytes and PAS-positive granular leukocytes (PAS-GL), when compared with values in control fish (Tavares-Dias et al. 2007). Freshwater fish body is hypertonic to the surrounding environment and so upon infection with *Saprolegnia* and subsequent skin damage this could results in influx of the water into fish body, potentially resulting in osmoregulatory failure and disruption in the electrolytes homeostasis (Tripathi et al. 2005).

## Conclusion

In conclusion, KMnO_4_ has proven a beneficial effect against saprolegniosis and show a protective role against oxidative damage in saprolegniosis-infected Nile tilapia while parenteral administration of adjuvant showed better fish survival rate in *Saprolegnia* infected Nile tilapia due to induction of innate immune response and enhancement of fish resistance against infection. Also, it showed the ability to offer a marked protective effect against oomycetes infection in farmed fish stocks

## Methods

### Chemical agents

#### Potassium permanganate (KMnO_4_)

Potassium permanganate (KMnO_4_); Nasr. Co., Cairo, Egypt. A stock solution of KMnO_4_ was prepared by dissolving 1 g of KMnO_4_ in 1 L of reagent grade water. KMnO_4_ dosed at 2.5 ppm (mg/l), calculated according to potassium permanganate demand average of the experimental tanks 0.5 mg/l +2 mg/l according to (Plumb and Hanson 2010).

#### Freund’s complete adjuvant (FCA)

Freund’s complete adjuvant (Difco #263810), consisting of 5 mg of killed, dessicated *Mycobacterium butyricum* in 10 ml of adjuvant (8.5 ml paraffin oil and 1.5 ml of manniden monooleate) was prepared immediately before use.

### Fungal strain

One isolate of *Saprolegnia* used in challenge experiment; *Saprolegnia ferax* isolate. It was isolated from skin lesions of Nile tilapia suffered saprolegniosis from different fish farms in Egypt. Isolates were identified according to their morphological and sexual character and sequenced (unpublished data). Fungal isolates were cultured on glucose yeast extract (GY) agar at 19°C. Agar with mycelia was then aseptically cut into 1 × 1 cm^2^ squares and placed into a Petri dish with 30 mL GY broth. After 2 days, the agar remnants were removed, and the growing mycelia were cut and washed repeatedly in sterilized tap water (TW) and then transferred into 20 mL fresh sterilized TW and kept for 18–24 h at 19°C (Kitancharoen and Hatai 1996). After the zoospores of the tested *Saprolegnia* strains were harvested; they were counted with the zoospore suspension was counted using a haemocytometer (Bürker Türk) and then added to experimental tanks at a concentration of 2 × 10^4^ zoospore/l.

### Pathogenicity experiment

A total of one hundred and sixty Nile tilapia (*O. niloticus*) weighed 70 g were placed to eight 60-l aquarium tanks with freshwater at 15°C. Fish were fed on commercial diet *ad libitum* and maintained under 12 h light/ 12 h dark photoperiod. Water quality during all experiments was: dissolved oxygen 6.8–7.5 mg/l, temperature 15°C, pH 6.65–6.87, unionized ammonia <0.001 mg/l and nitrite <0.10 mg/l. Fish were acclimated for 2 weeks. Fish experimental protocol and handling was performed with regard to the ethical committee of veterinary medicine faculty and local Mansoura university rules. The fish were placed 20 fish /aquarium in duplicate aquaria per each group (40 fish/group). The experimental groups were: control negative group without zoospores exposure; infected group exposed to 2 × 10^4^ zoospores of *S. ferax* per liter of water (spore/L) and KMnO_4_ group is the infected group treated after one week with 2.5 ppm of KMnO_4;_ by adding 150 mL of the stock solution to each treated tank, FCA group is the infected group treated after one week with intraperitoneal injection of 0.1 ml of FCA and both groups lasted for 2 weeks after, and control positive group exposed to 2 × 10^4^ spore/L for the 3 weeks experimental period. Fish in all groups were descaled on different regions on the body using sharp scalpel then, 2 × 10^4^ spore/L were added to all groups except the control negative one. All aquaria were covered to minimize contamination. Water changed once a week during the treatment period with replacing the concentration of the KMnO_4_ to be at the same exposure level for group 2. The aquaria were checked daily after the challenge for two weeks, and dead and moribund fish were removed for examination. Skin scrapings, gill and fin biopsies of removed fish were examined. *S. ferax* infection was confirmed via identification of broad aseptate hyphae, sporangia and encysted zoospores with light microscopy.

### Fungal diagnosis

The diagnosis of fungal infection was based on the appearance of cottony mycelial growth on the surface of the fish. *Saprolegnia* infection was identified by their morphological characters on Nile tilapia by direct microscopic examination of lesion and mycological culture on glucose yeast extract (GY) agar at 19°C. Identification was based on the classical morphological criteria of Seymour (1970) and Willoughby (1978, 1985). The numbers of fish mortalities, moribund and surviving fishes were recorded. The dead fish and controls were also collected and subjected to analysis.

### Oxidative enzymes and biochemical analysis

Four fish from each group (8 fish/group) were randomly selected at week 1, 2 and 3. Blood samples were collected from the caudal vein, left to coagulate, then centrifuged for 5 min at 3000 r/m for serum separation to be used for serum nitric oxide (NO), superoxide dismutase (SOD) and glutathione (GSH) measurement, moreover for some biochemical parameters measurement.

1. Oxidative enzymes (Nitric oxide, SOD and GSH):
  The serum NO, SOD and GSH were assayed spectrophotometrically (5010, Photometer, BM Co. Germany) using commercial test kits(Bio-Chain, Inc., USA).
2. Serum biochemical analysis:

Serum aspartate aminotranseferase (AST) and alanine aminoranferase (ALT) were estimated using commercial kits (Randox UK), also total protein, albumin and serum sodium (Na^+^) ,potassium (K^+^) levels were measured sepectrophotometrically using test kits (stanbio.), biodiagnostic respectively.

### Statistical analysis

Data were expressed as means standard errors. Statistical analysis was performed using the software SPSS 19 (SPSS Inc, Chicago, Illinois). All data were subjected to analyses other than mortality data, which was treated as frequency data. For all tests, the effect of experimental variables (aquarium replicate, sampling day) was tested by analysis of variance One-way ANOVA and Duncan’s multiple comparisons of the means to compare data obtained. Differences between treatments were considered significant when P < 0.05.

## Authors’ original submitted files for images

Below are the links to the authors’ original submitted files for images. Authors’ original file for figure 1 Authors’ original file for figure 2 Authors’ original file for figure 3 Authors’ original file for figure 4 Authors’ original file for figure 5

## Abbreviations

S. ferax: *Saprolegnia. ferax*
KMnO4: Potassium permanganate
FCA: Freund’s complete adjuvant
NO: Nitric oxide
GSH: Glutathione
SOD: Superoxide dismutase
AST: Aspartate aminotranseferase
ALT: Alanine aminoranferase
Na+: Sodium
K+: Potassium
ROS: Reactive oxygen species.
