# Supplementary figures and images for: Protective role of adjuvant and potassium permanganate on oxidative stress response of Nile tilapia (Oreochromis niloticus) challenged with Saprolegnia ferax
Source: Springerplus. 2013 Mar 9;2(1):94. doi: 10.1186/2193-1801-2-94 (PMC3647092; doi:10.1186/2193-1801-2-94)

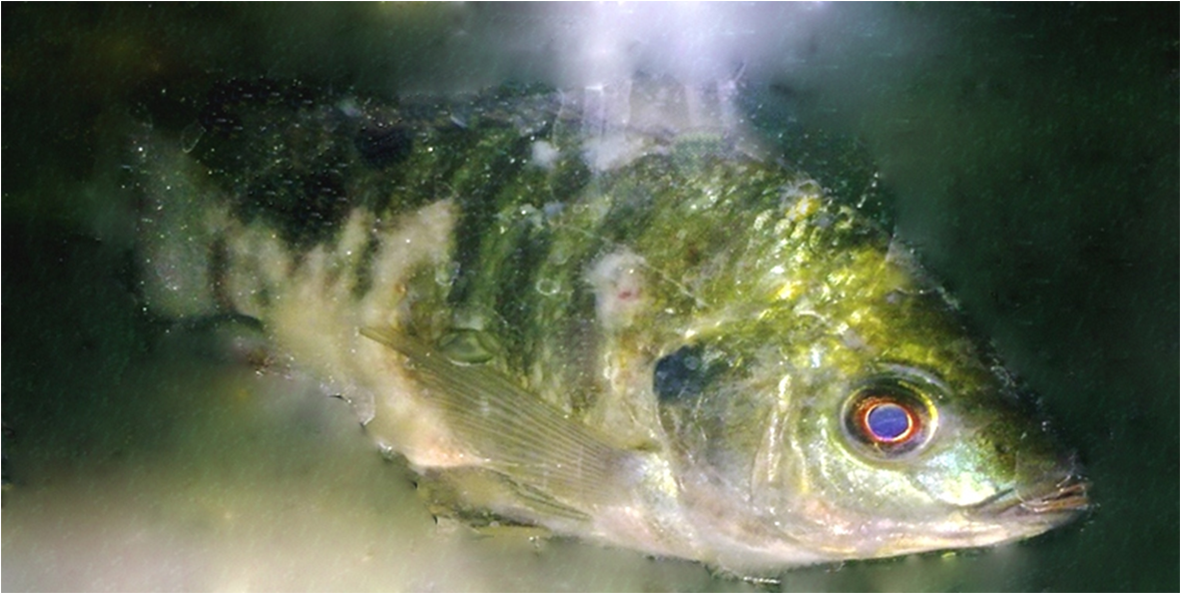

Supplement: Supplementary file 1 — Authors’ original file for figure 1 [file 40064_2013_223_MOESM1_ESM.tiff]

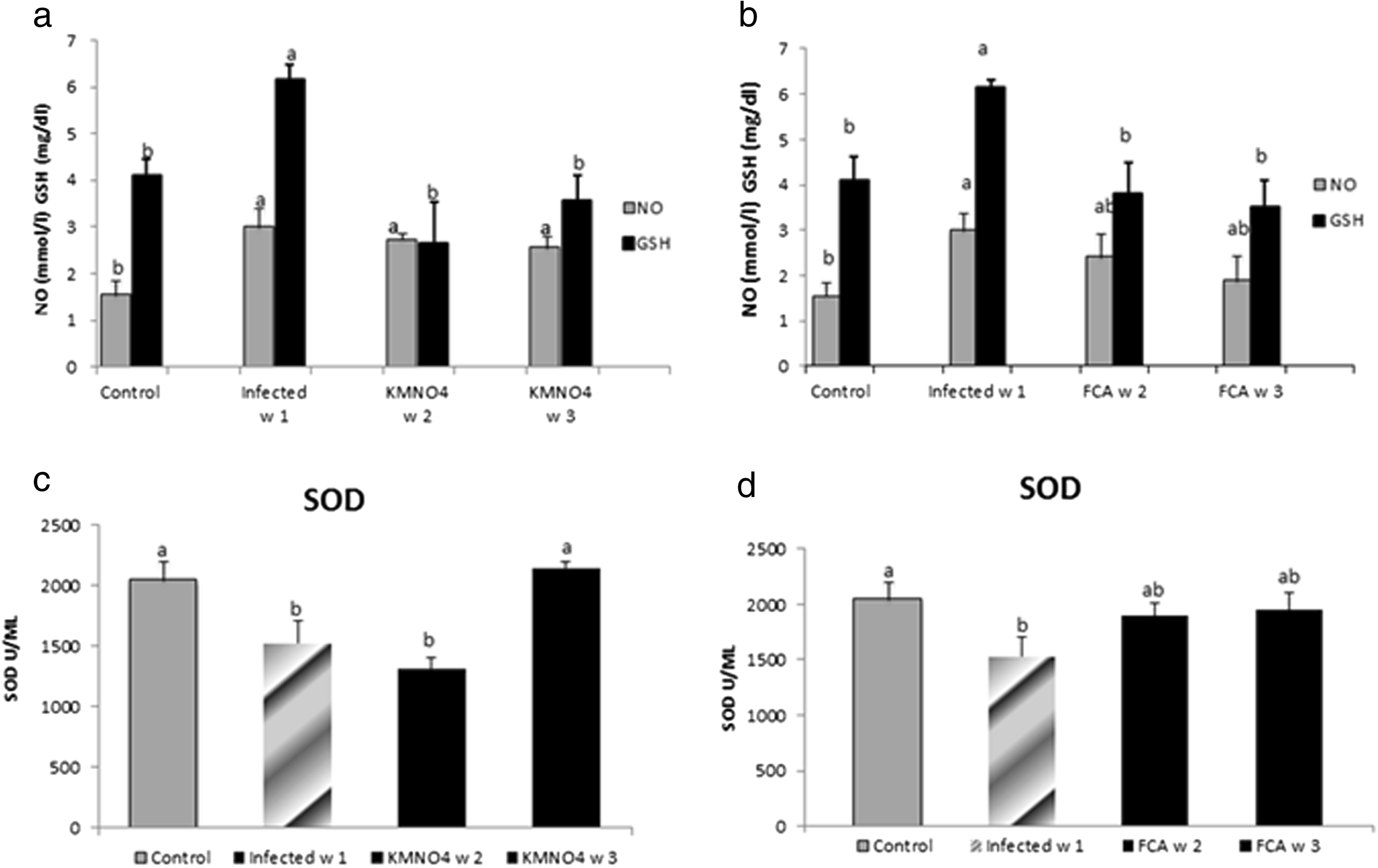

Supplement: Supplementary file 2 — Authors’ original file for figure 2 [file 40064_2013_223_MOESM2_ESM.tiff]

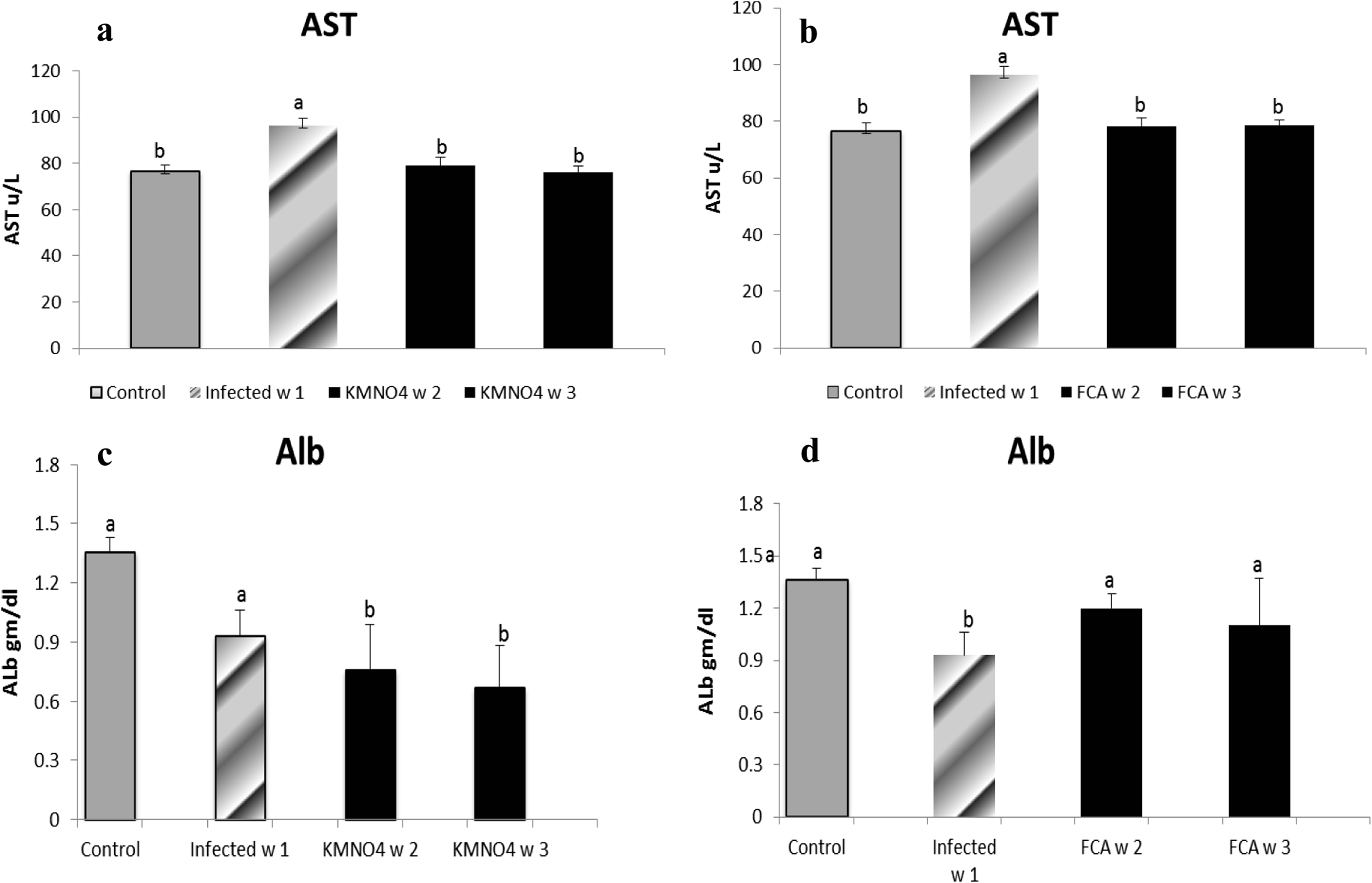

Supplement: Supplementary file 3 — Authors’ original file for figure 3 [file 40064_2013_223_MOESM3_ESM.tiff]

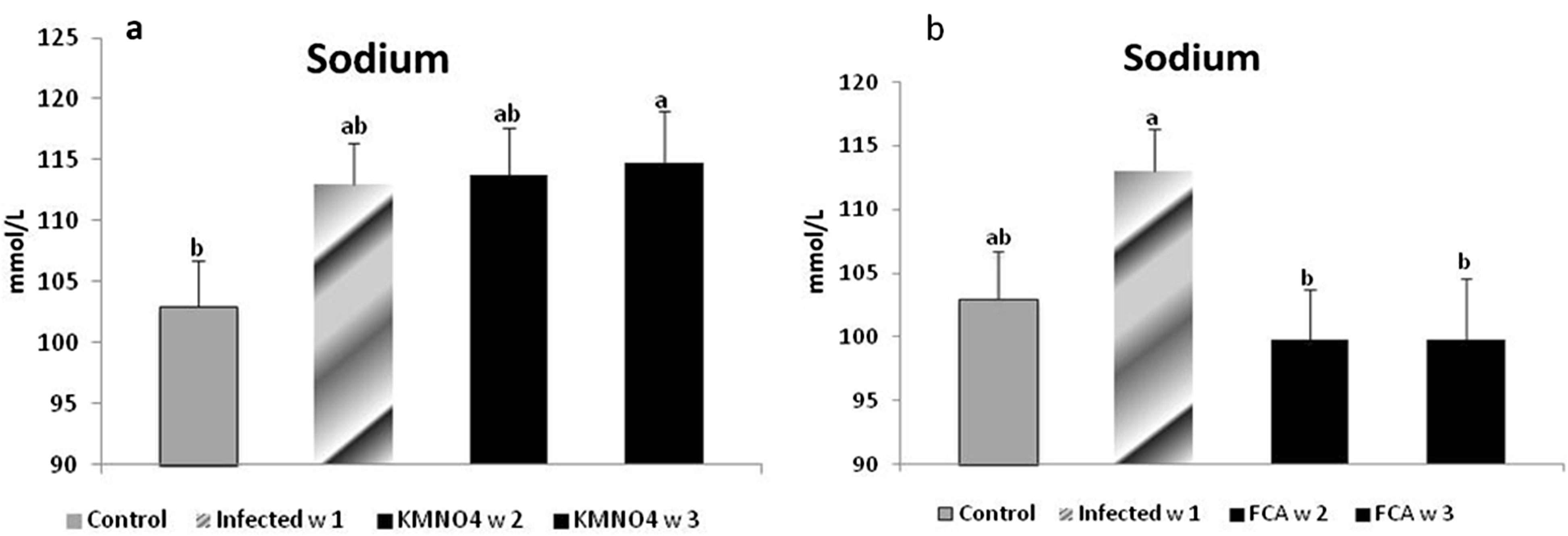

Supplement: Supplementary file 4 — Authors’ original file for figure 4 [file 40064_2013_223_MOESM4_ESM.tiff]

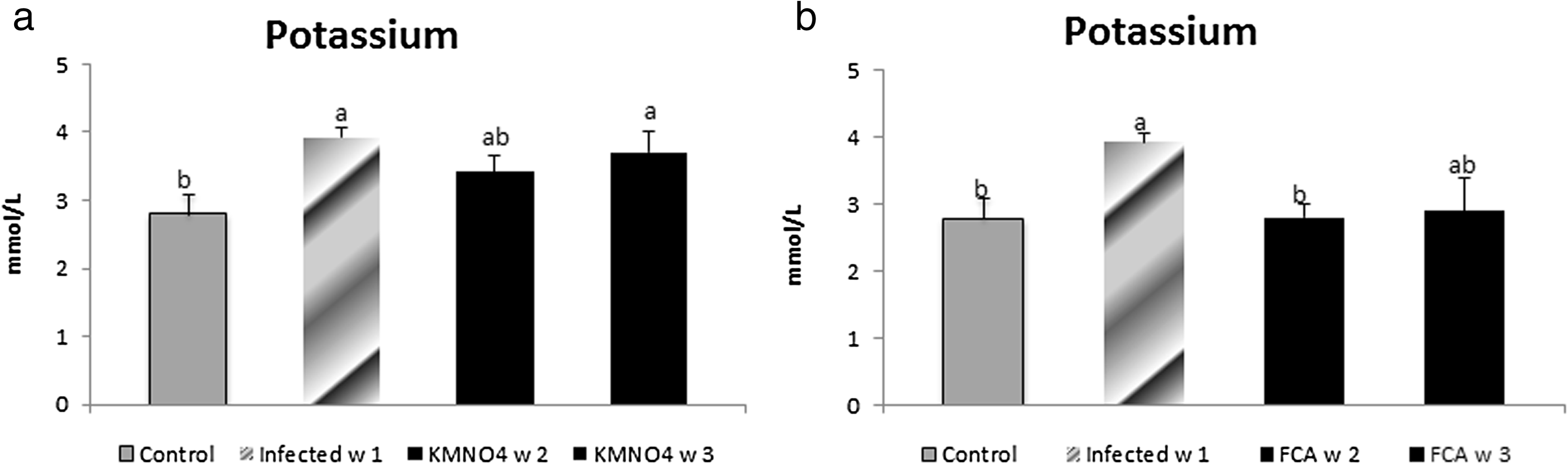

Supplement: Supplementary file 5 — Authors’ original file for figure 5 [file 40064_2013_223_MOESM5_ESM.tiff]
